# Lack of Association between Serum Vitamin B_6_, Vitamin B_12_, and Vitamin D Levels with Different Types of Glaucoma: A Systematic Review and Meta-Analysis

**DOI:** 10.3390/nu9060636

**Published:** 2017-06-21

**Authors:** Shengjie Li, Danhui Li, Mingxi Shao, Wenjun Cao, Xinghuai Sun

**Affiliations:** 1 Department of Clinical Laboratory, Eye & ENT Hospital, Shanghai Medical College, Fudan University, Shanghai 200031, China; lishengjie6363020@163.com (S.L.); smingxi@sohu.com (M.S.); 2 Key Laboratory of Environment and Genes Related to Diseases, Ministry of Education, Medical School, Xi’an Jiaotong University, Xi’an 710061, China; danhuili1002@sina.com; 3 Department of Ophthalmology & Visual Science, Eye & ENT Hospital, Shanghai Medical College, Fudan University, Shanghai 200031, China; 4 State Key Laboratory of Medical Neurobiology, Institutes of Brain Science and Collaborative Innovation Center for Brain Science, Fudan University, Shanghai 200031, China; 5 Key Laboratory of Myopia, Ministry of Health, Fudan University, Shanghai 200031, China; 6 Shanghai Key Laboratory of Visual Impairment and Restoration, Fudan University, Shanghai 200031, China

**Keywords:** glaucoma, vitamin B_6_, vitamin B_12_, vitamin D, meta-analysis

## Abstract

Although vitamins play a major role in health, and their deficiency may be linked to symptoms of optic-nerve dysfunction, the association between serum vitamin levels and glaucoma in humans remains controversial. In this study, articles in the PubMed, Web of Science, and EMBASE databases were searched up to 25March 2017. Nine studies on primary open-angle glaucoma (POAG), four studies on normal tension glaucoma (NTG), and six studies on exfoliative glaucoma (EXG) were retrieved. The combined results showed no differences in the levels of serum vitamin B_6_ between POAG (*p* = 0.406) and EXG (*p* = 0.139) patients and controls. The weighted mean differences (WMDs) with 95% confidence intervals (CIs) were 2.792 ng/mL (−3.793 to 9.377) and 1.342 ng/mL (−3.120 to 0.436), respectively. There was no difference between POAG (*p* = 0.952), NTG (*p* = 0.757), or EXG (*p* = 0.064) patients and controls in terms of serum vitamin B_12_. The WMDs with 95% CIs were 0.933 pg/mL (−31.116 to 29.249), 6.652 pg/mL (−35.473 to 48.777), and 49.946 pg/mL (−102.892 to 3.001), respectively. The serum vitamin D levels exhibited no differences (*p* = 0.064) between POAG patients and controls; the WMD with 95% CI was 2.488 ng/mL (−5.120 to 0.145). In conclusion, there was no association found between serum vitamin B_6_, vitamin B_12_, or vitamin D levels and the different types of glaucoma.

## 1. Introduction

Glaucoma is the second leading cause of irreversible blindness worldwide; its prevalence is expected to increase from 60.5 million people in 2010 to 79.6 million by 2020 [1]. The common characteristic features of glaucoma are retinal ganglion cell apoptosis and visual field changes [2]. There are two types of adult-onset glaucoma, as follows: open-angle glaucoma (OAG), in which the angle of the anterior chamber is anatomically open, and angle-closure glaucoma, in which the angle is anatomically closed. OAG is the most common type of glaucoma; based on the intraocular pressure (IOP), it is divided into primary open-angle glaucoma (POAG; IOP > 21 mm Hg) and normal tension glaucoma (NTG; untreated IOP ≤ 21 mm Hg). Exfoliative glaucoma (EXG) is characterized by high IOP and worse 24-h IOP, and it represents the most common type of secondary OAG. Various risk factors have been investigated to evaluate the associations with the development and progression of glaucoma, including increased age, gender, high IOP, and genetic variants [3,4]. However, the precise mechanisms involved in glaucoma are yet to be determined.

Recently, Williams et al. [5] reported that vitamin B_3_ can modulate mitochondrial vulnerability and prevent glaucoma in mice. Although many publications have reported a difference in serum vitamin levels between patients with glaucoma and normal subjects, the association between serum vitamin levels and glaucoma in humans remains controversial. Turgut et al. [6] showed that the serum vitamin B_6_ levels are significantly higher in POAG and NTG patients than in controls, but others reported no significant difference in serum vitamin B_6_ levels among these groups [7,8,9]. Several studies have shown that serum vitamin B_12_ levels are elevated in NTG, POAG, and EXG [6,10,11], but others have reported decreased levels of serum vitamin B_12_ [9,12]. Moreover, although most studies have found that the serum vitamin D level is decreased in glaucoma patients compared with controls [13,14], the difference in vitamin D levels between glaucoma patients and controls is limited.

Vitamins are dietary components that are required for the proper function of the methylation cycle, monoamine oxidase production, DNA synthesis, and phospholipid repair and maintenance [15,16]. They play a major role in health, and their deficiencies may be linked to symptoms of neuronal dysfunction. However, the number of published papers evaluating the associations between serum vitamin (A, C, E) levels and different types of glaucoma is relatively limited. Therefore, we aim to perform a systematic review and meta-analysis by combining individual studies and summarizing an overall effect size for the association between vitamin B_6_, vitamin B_12_, and vitamin D levels with different types of glaucoma.

## 2. Methods

### 2.1. Publication Search

Eligible articles were aggregated from three databases, namely PubMed, Web of Science, and EMBASE; these were published in the English language from 1 January 1990 to 25 March 2017. The following search terms were used: “glaucoma” (in the title) and “vitamin” (in the title/abstract). Moreover, a manual search was performed by checking the reference lists of the reports on clinical trials, meta-analyses, and systematic reviews that were examined. Two reviewers (Shengjie Li and Danhui Li) completed the literature search independently.

### 2.2. Inclusion Criteria

All studies had to meet the following inclusion criteria:

(1) The investigation involved random sampling or cluster sampling;
(2) Two or more comparison groups (glaucoma and control groups) were included;
(3) Healthy subjects were recruited for the control group;
(4) A laboratory assessment of serum or plasma vitamin levels (vitamin B_6_/vitamin B_12_/vitamin D) was conducted;
(5) The study was published in English;
(6) The full text of the article was accessible; and
(7) The subjects were human.

### 2.3. Study Selection and Data Extraction

Study selection was performed by two independent investigators (Shengjie Li and Danhui Li) according to the inclusion criteria listed. From each study, the following data were collected and reviewed independently by the two investigators (Shengjie Li and Danhui Li): the first author’s name, country/region, publication year, mean age of participants, sample size, type of glaucoma, and considered vitamin (vitamin B_6_/vitamin B_12_/vitamin D). Moreover, we conducted a focused discussion to resolve any disagreements.

### 2.4. Quality Assessment

The quality assessments for the study were based on an examination of the previously reported guidelines for glaucoma studies [17,18]. We developed a quality score for each included study that was reviewed independently by two investigators (Shengjie Li and Danhui Li) evaluating six items, as follows:

(1) Was the study design clearly described?
(2) Were the diagnostic criteria and clinical examinations comprehensive and standardized?
(3) Were the participant selection procedures reported clearly?
(4) Was the participant enrollment duration provided?
(5) Were the age and sex of eligible participants clearly described?
(6) Were the serum vitamin B_6_/B_12_/D measurement methods clearly reported?

In the scoring system, for each quality item, a response of “clear or adequate” resulted in a score of 1 point, whereas a response of “no” received a score of 0 points. The study was considered as being of adequate quality if the quality score was greater than or equal to 4. Studies of inadequate quality were excluded from this meta-analysis.

### 2.5. Statistical Analysis

The weighted mean differences (WMDs) in vitamin B_6_, vitamin B_12_, and vitamin D levels between glaucoma (POAG/NTG/EXG) and control groups and 95% confidence intervals (CIs) were calculated for each study. The heterogeneity of the pooled studies was estimated using the χ^2^-based Q statistic and *I*^2^ metrics. A random-effects model was used if heterogeneity was observed (*p* < 0.05 or *I*^2^ > 60%); otherwise, a fixed-effects model was applied. A funnel plot analysis and Egger’s test were performed to assess potential publication bias. We performed a sensitivity analysis to evaluate the stability of the results through the leave-one-out strategy. This method uses the sequential omission of individual studies in every comparison to determine whether there is a significant alteration of the combined values. A value of *p* < 0.05 was considered statistically significant. The statistical analyses were performed using Comprehensive Meta-Analysis version 2.0 (Biostat, Englewood Cliffs, NJ, USA).

## 3. Results

### 3.1. Search Results and Study Characteristics

**Figure 1:**
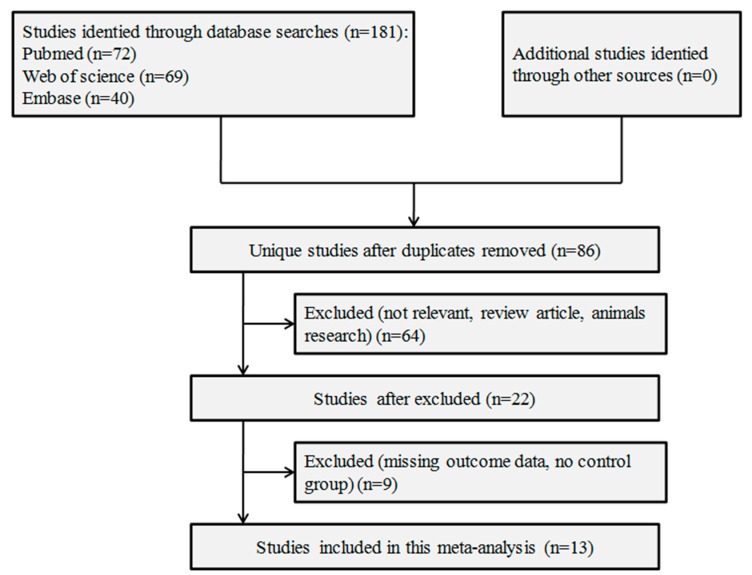
Study selection flow chart.

A flowchart illustrating the article search process is presented in Figure 1. The initial search strategy identified 72 studies in PubMed, 69 in Web of Science, and 40 in EMBASE. From the 181 studies, 168 were excluded. Finally, 13 studies [6,7,8,9,10,11,12,13,14,19,20,21,22] were included in this meta-analysis. The detailed characteristics of each included study are presented in Table 1. The quality scores of these studies ranged from 4 to 6, with a mean of 5.08. Detailed scoring results are presented in Table 2.

According to the type of glaucoma, 13 studies were categorized into three groups. Since some studies discussed different types of glaucoma, they could be included in more than one group. Thus, there were nine studies included in the POAG group [6,7,8,10,11,12,13,14,19], four in the NTG group [6,9,10,12], and six in the EXG group [6,11,12,20,21,22]. Moreover, according to the different types of vitamins considered (vitamin B_6_/vitamin B_12_/vitamin D), the following categorizations were identified:

(1) The POAG group: Three studies considered vitamin B_6_ (109 cases and 115 controls), six considered vitamin B_12_ (222 cases and 249 controls), and three considered vitamin D (513 cases and 5629 controls);
(2) The NTG group: Two studies considered vitamin B_6_ (90 cases and 82 controls), four considered vitamin B_12_ (123 cases and 176 controls), and 0 considered vitamin D; and
(3) The EXG group: Three studies considered vitamin B_6_ (144 cases and 146 controls), six considered B_12_ (228 cases and 240 controls), and one considered vitamin D (70 cases and 70 controls).

### 3.2. Meta-Analysis of the Association of Vitamin B_6_ with POAG and EXG

**Figure 2:**
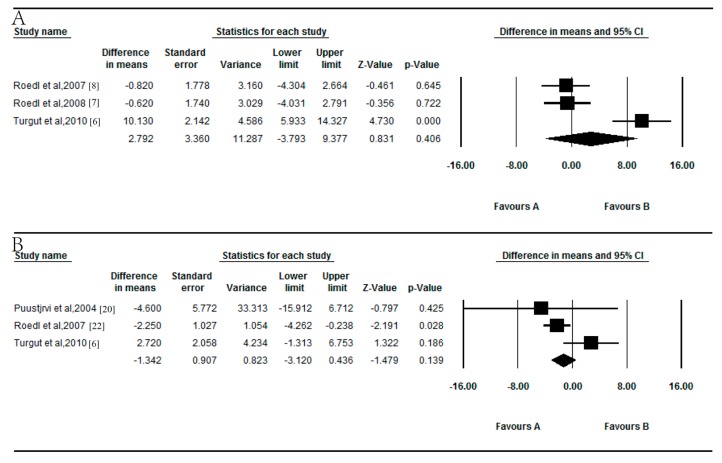
Forest plot of random effects meta-analysis showing the association of serum vitamin B_6_ levels with POAG (**A**) and EXG (**B**).

The combined results showed no difference in the serum vitamin B_6_ levels between POAG patients and controls (*p* = 0.406; Figure 2A). The WMD was 2.792 ng/mL (95% CI = −3.793 to 9.377), and there was significant between-study heterogeneity for vitamin B_6_ among the available studies (*p* < 0.0001, *I*^2^ = 89.581%). Moreover, there was no significant difference in serum vitamin B_6_ between EXG patients and controls (*p* = 0.139; Figure 2B). The WMD was 1.342 ng/mL (95% CI = −3.120 to 0.436), and there was no significant heterogeneity for vitamin B_6_ between the available studies (*p* = 0.082, *I*^2^ = 59.982%).

### 3.3. Meta-Analysis of the Association of Vitamin B_12_ with POAG, NTG, and EXG

**Figure 3:**
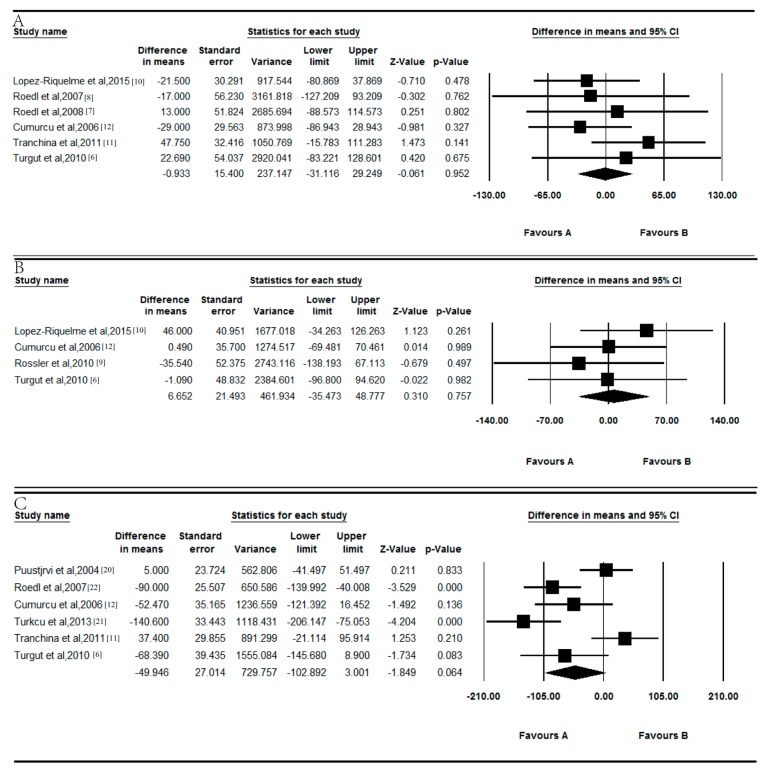
Forest plot of random effects meta-analysis showing the association of serum vitamin B_12_ levels with POAG (**A**), NTG (**B**), and EXG (**C**).

In the combined results, the levels of serum vitamin B_12_ exhibited no difference between POAG patients and controls (*p* = 0.952; Figure 3A). The WMD was 0.933 pg/mL (95% CI = −31.116 to 29.249), and there was no significant between-study heterogeneity for vitamin B_12_ among the available studies (*p* = 0.555, *I*^2^ = 0.000%).

The level of serum vitamin B_12_ showed no significant difference between NTG patients and controls (*p* = 0.757; Figure 3B). The WMD was 6.652 pg/mL (95% CI = −35.473 to 48.777), and there was no significant between-study heterogeneity for vitamin B_12_ among the available studies (*p* = 0.653, *I*^2^ = 0.000%). Moreover, there was no significant difference in serum vitamin B_12_ between EXG patients and controls (*p* = 0.064; Figure 3C). The WMD was 49.946 pg/mL (95% CI = −102.892 to 3.001), and there was significant between-study heterogeneity for vitamin B_12_ among the available studies (*p* < 0.001, *I*^2^ = 78.935%).

### 3.4. Meta-Analysis of the Association between Vitamin D and POAG

**Figure 4:**
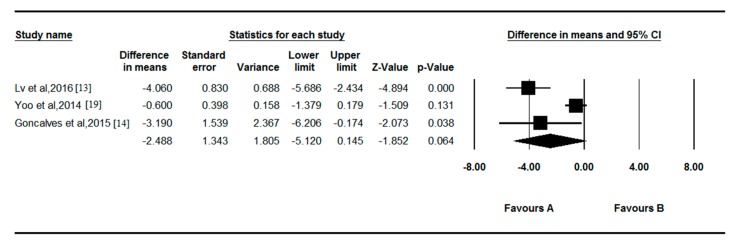
Forest plot of random effects meta-analysis showing the association between serum vitamin D and POAG.

The combined results showed that there was no significant difference in serum vitamin D levels between POAG patients and controls (*p* = 0.064; Figure 4). The WMD was 2.488 ng/mL (95% CI = −5.120 to 0.145), and there was significant between-study heterogeneity for vitamin D among the available studies (*p* < 0.001, *I*^2^ = 87.229%).

### 3.5. Analysis of Publication Bias

**Figure 5:**
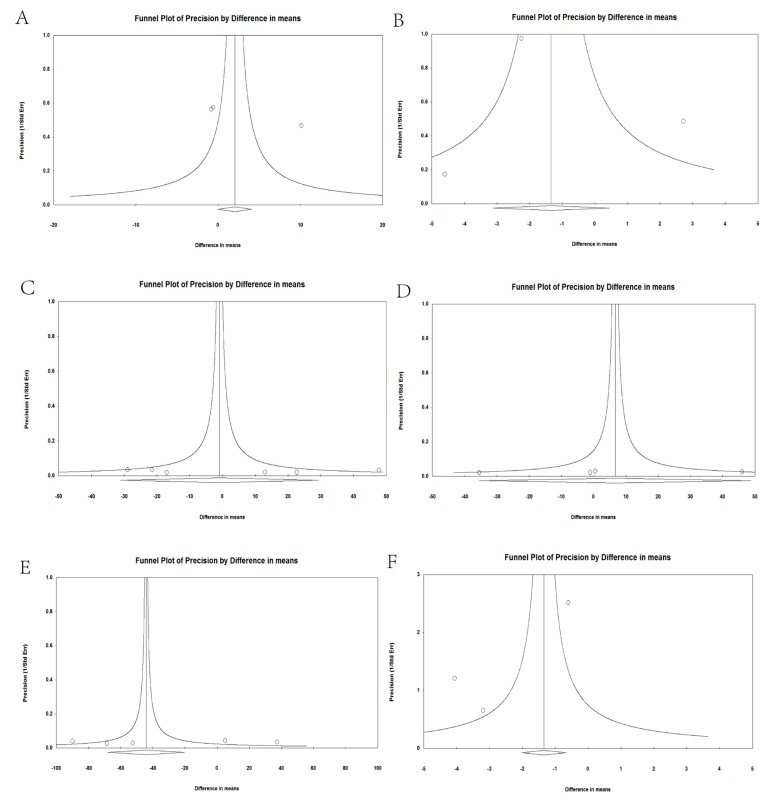
Funnel plot analysis to detect publication bias, vitamin B_6_ in POAG (**A**), vitamin B_6_ in EXG (**B**), vitamin B_12_ in POAG (**C**), vitamin B_12_ in NTG (**D**), vitamin B_12_ in EXG (**E**), and vitamin D in POAG (**F**).

There were no obvious asymmetries in the funnel plots. The *p*-values exceeded 0.05 for the following groups: vitamin B_6_ in POAG (*t* = 3.369, *p* = 0.07, 95% CI = −13.19 to 69.22; Figure 5A), vitamin B_6_ in EXG (*t* = 0.271, *p* = 0.83, 95% CI = −29.92 to 31.22; Figure 5B), vitamin B_12_ in POAG (*t* = 0.463, *p* = 0.67, 95% CI = −3.43 to 4.80; Figure 5C), vitamin B_12_ in NTG (*t* = 0.732, *p* = 0.54, 95% CI = −13.44 to 9.53; Figure 5D), vitamin B_12_ in EXG (*t* = 0.271, *p* = 0.83, 95% CI = −29.92 to 31.22; Figure 5E), and vitamin D in POAG (*t* = 1.332, *p* = 0.41, 95% CI = −40.72 to 33.00; Figure 5F).

### 3.6. Sensitivity Analysis

In the meta-analysis of the association between serum vitamin levels and the different types of glaucoma (POAG/NTG/EXG), the sensitivity analysis revealed that one study had a slight influence on the result; the results from the sensitivity analysis showed no associations of serum vitamin B_6_ levels with POAG, vitamin B_12_ with POAG, or vitamin B_12_ with NTG after this study was excluded (Table 3). Moreover, one study influenced the meta-analysis results regarding the association between vitamin B_6_ and EXG (Table 3), two studies influenced the results regarding the association between vitamin B_12_ and EXG (Table 3), and one study influenced the results regarding the association between vitamin D and POAG (Table 3).

## 4. Discussion

The relationship between serum vitamin levels and the presentation of NTG, POAG, or EXG remains uncertain; however, serum vitamin levels are considered to be associated with NTG, POAG, and EXG in terms of protecting neuronal function [23]. Thus, we performed the present meta-analysis to clarify this relationship. However, the meta-analysis results suggested that there is no evidence to confirm the association of serum vitamin levels with different types of glaucoma.

The exact biological mechanisms of the action of serum vitamins (vitamin B_6_/vitamin B_12_/vitamin D) in NTG, POAG, and EXG are not fully understood. Salari et al. [24] found that adding vitamin D to routine disease therapy had no significant effect on the thickness of the retinal nerve fiber layer or macula in patients with optic neuritis in a randomized, placebo-controlled trial study. In contrast, a recent article proposed that 1α,25-Dihydroxyvitamin D(3), or an analog thereof, may be used to treat glaucoma [25]. Romano et al. [26] suggested that vitamin B_12_ treatment represents a powerful strategy to accelerate not only re-epithelization, but also corneal re-innervation after mechanical injury. Contradictory results have also been reported for the association between serum vitamin B_12_ and glaucoma [6,9,10,11,12].

### 4.1. Vitamin B_6_ in POAG and EXG

Some researchers have suggested that vitamin B_6_ is associated with POAG and EXG. Turgut et al. [6] performed a case-control study and reported that the serum vitamin B_6_ levels were significantly increased in POAG and NTG patients. However, contrasting results, where in the vitamin B_6_ levels were not significantly different between POAG or EXG and controls, were also reported by Roedl et al. [7,8] and Rössler et al. [9]. In this subgroup meta-analysis, we did not detect any difference in serum vitamin B_6_ between POAG or EXG patients and controls. However, there was significant heterogeneity in POAG research concerning vitamin B_6_ among the available studies; such heterogeneity was not evident among EXG studies.

The significant heterogeneity concerning POAG research may be partially explained in terms of variances in nutritional status, diet, and lifestyle in the different populations studied (German vs. Turkish), as well as the vitamin B_6_ detection kits that were used (Bio-Rad, Munich, Germany vs. Shimadzu Corporation, Kyoto, Japan). In addition, the sensitivity analysis concerning serum vitamin B_6_ levels in patients with POAG indicated that the result was not greatly influenced by the exclusion of any individual study. However, the sensitivity analysis also showed that one study [6] influenced the meta-analysis results regarding the association between vitamin B_6_ and EXG.

### 4.2. Vitamin B_12_ in POAG, NTG, and EXG

Some studies have claimed that vitamin B_12_ is associated with POAG, NTG, and EXG. Turgut et al. [6], López-Riquelme et al. [10], and Tranchina et al. [11] suggested that glaucoma patients have higher serum vitamin B_12_ levels than normal controls. In contrast, two publications claimed that serum vitamin B_12_ levels were lower in individuals with glaucoma than in healthy controls [9,12]. The combined results from this meta-analysis showed that there was no difference in the levels of serum vitamin B_12_ between POAG, NTG, or EXG patients and controls (*p* = 0.952). However, significant heterogeneity in EXG studies on vitamin B_12_ was observed, and two studies influenced the meta-analysis results regarding the association between vitamin B_12_ and EXG. This may be partially explained by the variances in sample number (24 to 70). The different definitions of EXG and the difference in the vitamin B_12_ measurements may also have influenced the outcomes. At present, the most frequently used methods are a competitive chemiluminescent enzyme immunoassay [11,12,21], the time-resolved fluoroimmunoassay method [20], and an immunoassay [22]. However, these methods have not been standardized, so between-study comparisons are difficult. Furthermore, our sensitivity analysis indicated that the validity of the summary effect was stable in POAG and NTG studies, and this did not change materially when individual studies were excluded.

### 4.3. Vitamin D in POAG

No significant difference in serum vitamin D levels between POAG patients and controls was detected in this meta-analysis. However, there was significant between-study heterogeneity among the available studies on vitamin D, and one study influenced the meta-analysis results regarding the association between vitamin D and POAG. The reason for this maybe that one included study was a cross-sectional study, while the other two were case-control studies. Moreover, significant differences in the sample size and vitamin D measurements (enzyme-linked immunosorbent assay vs. radioimmunoassay) were detected, which may have also influenced the outcome. A randomized trial with vitamin D supplementation may be more valuable to evaluate the temporal and causal relationship between vitamin D and glaucoma risk.

Although a standard search strategy and a thorough computerized search method were applied, certain limitations of our meta-analysis should be considered. First, the studies differed widely in terms of the study populations’ characteristics and measurement techniques. Second, although the quality scores of the studies ranged from 4 to 6, representing high quality data, the studies included in this meta-analysis were often small-scale, single-center studies.

## 5. Conclusions

In this meta-analysis, we reported that there is no association between serum vitamin B_6_, vitamin B_12_, and vitamin D levels with different types of glaucoma. However, it seems a little early to draw a conclusion based on the limited number of available studies so far. Consequently, a focus on the possible role of vitamins in the pathogenesis of different types of glaucoma may be highly desirable in future research. Therefore, a forward-looking, multi-center study with a larger sample size ought to be conducted.

## Figures and Tables

**Table 1:** Characteristics of Studies of Vitamin B_6_, Vitamin B_12_, and Vitamin D with Different Types of Glaucoma.

First Author	Year	Country	Glaucoma Group					Control Group				
			No.	Age	Vitamin B_6_ ng/mL	Vitamin B_12_ pg/mL	Vitamin D ng/mL	No.	Age	Vitamin B_6_ ng/mL	Vitamin B_12_ pg/mL	Vitamin D ng/mL
**POAG**												
Lv et al. [13]	2016	China	73	61.03 ± 2.75			26.37 ± 5.83	71	60.14 ± 3.03			30.43 ± 3.91
López-Riquelme et al. [10]	2015	Spain	48	50.0 ± 9.4		404.2 ± 198.2		75	43.7 ± 12.4		425.7 ± 137.7	
Roedl et al. [8]	2007	Germany	39	69.3 ± 8.4	12.64 ± 6.50	461.7 ± 228.9		39	70.5 ± 10.7	13.46 ± 9.00	478.7 ± 266.3	
Roedl et al. [7]	2008	Germany	36	67.3 ± 8.2	12.50 ± 7.15	438 ± 243		36	68.5 ± 9.8	13.12 ± 7.61	425 ± 194	
Yoo et al. [19]	2014	Korea	290	63.3 ± 10.7			18.1 ± 6.5	5394	60.4 ± 10.1			18.7 ± 6.6
Goncalves et al. [14]	2015	France	150	73.0 ± 7.9			21.05 ± 12.61	164	75.1 ± 8.5			24.24 ± 14.48
Cumurcu et al. [12]	2006	Turkey	25	56.76 ± 12.58		232.84 ± 67.55		19	55.63 ± 4.04		261.84 ± 126.22	
Tranchina et al. [11]	2011	Italy	40	68.71 ± 8.65		444.9 ± 167.17		40	69.23 ± 7.21		397.15 ± 118.68	
Turgut et al. [6]	2010	Turkey	34	58 ± 7.5	30.22 ± 12.15	368.24 ± 262.65		40	62 ± 8.1	20.09 ± 5.54	345.55 ± 201.75	
**NTG**												
López-Riquelme et al. [10]	2015	Spain	15	45.3 ± 12.1		471.7 ± 177.6		75	43.7 ± 12.4		425.7 ± 137.7	
Cumurcu et al. [12]	2006	Turkey	18	57.77 ± 7.27		262.33 ± 85.94		19	55.63 ± 4.04		261.84 ± 126.22	
Rössler et al. [9]	2010	Germany	42	65.5 ± 12.1	14.45 ± 12.89	387.73 ± 282.04		42	63.1 ± 11.5	13.57 ± 10.41	423.27 ± 188.85	
Turgut et al. [6]	2010	Turkey	48	56 ± 6.8	30.50 ± 11.29	344.46 ± 247.84		40	62 ± 8.1	20.09 ± 5.54	345.55 ± 201.75	
**EXG**												
Puustjrvi et al. [20]	2004	Finland	36	77.4 ± 6.0	33.3 ± 20.1	313 ± 106		36	77.2 ± 5.4	37.9 ± 28.2	308 ± 95	
Roedl et al. [22]	2007	Germany	70	70.3 ± 8.2	10.29 ± 5.73	323 ± 129	18.1 ± 6.5	70	68.4 ± 11.6	12.54 ± 6.40	413 ± 170	18.7 ± 6.6
Cumurcu et al. [12]	2006	Turkey	24	61.66 ± 10.05		209.37 ± 104.44		19	55.63 ± 4.04		261.84 ± 126.22	
Turkcu et al. [21]	2013	Turkey	24	67.0 ± 6.9		232.2 ± 104.8		35	69.6 ± 6.5		372.8 ± 138.8	
Tranchina et al. [11]	2011	Italy	36	69.58 ± 5.92		434.55 ± 141.46		40	69.23 ± 7.21		397.15 ± 118.68	
Turgut et al. [6]	2010	Turkey	38	63 ± 6.3	22.81 ± 11.71	277.16 ± 139.08		40	62 ± 8.1	20.09 ± 5.54	345.55 ± 201.75	

**Table 2:** Quality Scores of Individual Studies.

	Components of the Quality Score						
First Author	(1)	(2)	(3)	(4)	(5)	(6)	Total
Lv et al. [13]	1	1	1	1	1	1	6
López-Riquelme et al. [10]	1	1	1	0	1	1	5
Roedl et al. [8]	1	0	0	1	1	1	4
Roedl et al. [7]	1	0	1	1	1	1	5
Yoo et al. [19]	1	1	1	1	1	1	6
Goncalves et al. [14]	1	1	1	1	1	1	6
Cumurcu et al. [12]	1	1	0	1	1	1	5
Tranchina et al. [11]	1	0	1	1	1	1	5
Turgut et al. [6]	1	1	0	0	1	1	4
Rossler et al. [9]	1	1	1	0	1	1	5
Roedl et al. [22]	1	1	0	0	1	1	4
Turkcu et al. [21]	1	1	1	0	1	1	5
Puustjrvi et al. [20]	1	1	1	1	1	1	6

Note: (1) Was study design clearly described? (2) Were diagnostic criteria and clinical examinations comprehensive and standardized? (3) Were participant selection procedures reported clearly? (4) Was participant enrollment duration provided? (5) Were the age and sex of eligible participants clearly described? (6) Were vitamin B_6_/B_12_/D measurement methods clearly reported?

**Table 3:** Sensitivity Analysis Using the Leave-one-out Strategy.

Study Omitted	Point	95% CI	*p*-Value
POAG (vitamin B_6_)			
Roedl et al., 2007 [8]	4.683	−5.851–15.216	0.384
Roedl et al., 2008 [7]	4.590	−6.140–15.320	0.402
Turgut et al., 2010 [6]	1.244	−3.155–1.720	0.564
EXG (vitamin B_6_)			
Puustjrvi et al., 2004 [20]	−0.130	−0.397–0.138	0.341
Roedl et al., 2007 [22]	0.065	−0.257–0.386	0.693
Turgut et al., 2010 [6]	−0.308	−0.579–0.037	0.026 *
POAG (vitamin B_12_)			
López-Riquelme et al., 2015 [10]	0.056	−0.155–0.267	0.602
Roedl et al., 2007 [8]	0.024	−0.176–0.224	0.811
Roedl et al., 2008 [7]	−0.001	−0.199–0.198	0.995
Cumurcu et al., 2006 [12]	0.040	−0.151–0.232	0.682
Tranchina et al., 2011 [11]	−0.057	−0.258–0.143	0.575
Turgut et al., 2010 [6]	−0.008	−0.207–0.191	0.936
NTG (vitamin B_12_)			
López-Riquelme et al., 2015 [10]	−0.061	−0.333–0.211	0.661
Cumurcu et al., 2006 [12]	0.013	−0.250–0.277	0.921
Rossler et al., 2010 [9]	0.089	−0.208–0.387	0.556
Turgut et al., 2010 [6]	0.021	−0.280–0.321	0.892
EXG (vitamin B_12_)			
Puustjrvi et al., 2004 [20]	−62.354	−121.997–2.711	0.040 ^Δ^
Roedl et al., 2007 [22]	−41.399	−102.731–19.333	0.186
Cumurcu et al., 2006 [12]	−49.770	−112.833–13.294	0.122
Turkcu et al., 2013 [21]	−32.109	−81.451–17.234	0.202
Tranchina et al., 2011 [11]	−67.396	−118.976–15.815	0.010 ^Δ^
Turgut et al., 2010 [6]	−46.934	−108.324–14.455	0.134
POAG (vitamin D)			
Lv et al., 2016 [13]	−1.468	−3.865–0.928	0.230
Yoo et al., 2014 [19]	−3.864	−5.295–2.433	<0.0001 ^Ω^
Goncalves et al., 2015 [14]	−2.253	−5.641–1.134	0.192

Note: CI = confience interval; Δ = influenced the meta-analysis results regarding the association between vitamin B_12_ and EXG; * = influenced the meta-analysis results regarding the association between vitamin B_6_ and EXG; ^Ω^ = influenced the meta-analysis results regarding the association between vitamin D and POAG.
